# Case report: Atypical anti-GlyR antibody-positive pediatric progressive encephalomyelitis with rigidity and myoclonus presenting as chronic joint pain

**DOI:** 10.3389/fimmu.2026.1941572

**Published:** 2026-09-22

**Authors:** Xiao-Xue Zou, Lian Meng, Jin-Yu Huang, Zhen-Fang Mao, Yun-Bo Zhang, Yang Yang, Wu-Xiao Wei

**Affiliations:** 1 Changde First Hospital of Traditional Chinese Medicine, Changde, Hunan, China; 2 The First Affiliated Hospital of Guangxi University of Science and Technology, Guangxi University of Science and Technology, Liuzhou, China; 3 Youjiang Medical University for Nationalities, Baise, China

**Keywords:** autoimmune disease, autoimmunity, children, glycine receptor, misdiagnosis, progressive encephalomyelitis with rigidity and myoclonus

## Abstract

Progressive encephalomyelitis with rigidity and myoclonus (PERM) represents the most severe subtype of stiff-person spectrum disorders and is exceedingly rare in children. We report a 14-year-old female patient who presented with chronic joint pain and axial rigidity as the initial manifestations, with a disease course of two years, during which she had been diagnosed with juvenile idiopathic arthritis but lacked typical peripheral arthritis signs and showed poor response to therapy. The patient exhibited paroxysmal axial rigidity, autonomic dysfunction, and sleep disturbances. Although typical brainstem signs were absent, serum anti-glycine receptor (GlyR) antibodies were positive at a titer of 1:100, and brain magnetic resonance imaging revealed a hyperintense lesion on T2-weighted imaging in the right brainstem, leading to a clinical diagnosis of anti-GlyR antibody-positive PERM. Following treatment with intravenous immunoglobulin (IVIG) combined with methylprednisolone, her symptoms resolved, and she remained relapse-free during one year of follow-up. This case highlights that PERM can present with chronic joint pain and axial rigidity as prominent initial manifestations in children. For patients with unexplained axial rigidity accompanied by autonomic symptoms, even in the absence of typical brainstem signs, early serum anti-GlyR antibody screening should be considered. Delayed diagnosis does not necessarily predict poor prognosis, and prompt immunomodulatory therapy can still achieve favorable long-term outcomes.

## Introduction

Progressive encephalomyelitis with rigidity and myoclonus (PERM) is a rare and potentially fatal autoimmune disorder of the central nervous system, representing the most severe clinical subtype within the stiff-person spectrum disorders (1). PERM is a brainstem, autonomic, and long-tract disorder characterized clinically by truncal and limb rigidity and painful spasms, often accompanied by hyperekplexia, oculomotor disturbances, dysphagia, and respiratory insufficiency, with a typically subacute or acute onset and a relapsing-remitting or progressive course (2, 3). Antibodies against the glycine receptor (GlyR) represent the most characteristic pathogenic biomarker of PERM (4). Since Carvajal-González et al. (2014) systematically reported the prevalence and pathogenicity of GlyR antibodies in PERM, GlyR antibody testing has been widely incorporated into the diagnostic workup of this condition (5). PERM is exceedingly rare in the pediatric population, and the first global case of anti-GlyR antibody-positive PERM in a child was not reported until 2013 by Damásio et al. (6). PERM is clinically rare and frequently presents with non-specific early symptoms, rendering it highly susceptible to misdiagnosis or delayed diagnosis; in severe cases, it may progress to respiratory failure and become life-threatening (7). Herein, we report a pediatric case of PERM characterized by an initial presentation of chronic joint stiffness and pain, with positive serum anti-GlyR antibodies but negative cerebrospinal fluid findings, and a diagnostic delay of up to two years from symptom onset. This case illustrates that PERM can present with an atypical, chronic, and predominantly musculoskeletal phenotype in children, and aims to expand the understanding of the heterogeneous clinical spectrum of pediatric PERM and to provide references for clinicians in the early recognition, diagnosis, and management of this potentially fatal disease.

## Case presentation

A 14-year-old female patient was admitted with a progressive two-year history of generalized rigidity and joint pain, with axial rigidity predominating over peripheral joint involvement. The symptoms had begun insidiously without identifiable precipitating factors, initially involving the bilateral shoulder joints and subsequently extending to the elbows, wrists, knees, and the cervical and thoracic spine, accompanied by intermittent headache and blurred vision. The episodes predominantly affected axial muscles, each lasting approximately 10 to 20 minutes with spontaneous resolution, recurring several times daily. During frequent episodes, the patient was unable to ambulate independently. The episodes were characterized by brief, involuntary, non-rhythmic jerking movements predominantly involving the trunk and proximal limbs, which could be triggered by auditory or tactile stimuli or occur spontaneously. The clinical course was also complicated by sleep disturbance, difficulty in turning over and combing her hair, as well as xerostomia and hyperhidrosis. Over the two years prior to admission, she had visited other hospitals and received a diagnosis of “juvenile idiopathic arthritis,” for which she underwent corresponding treatments; however, her response was poor, with persistent and progressive worsening of symptoms. The patient was the first child of non-consanguineous parents, with normal growth and developmental milestones, no family history of neurological disorders, and no history of long-term medication use. On admission, she presented with an abnormal posture and mild scoliosis. Her voice was low and weak, without dysarthria. Muscle tone in the limbs and trunk was markedly increased, with pronounced paroxysmal rigidity, and extensive tenderness and percussion pain were noted over the limbs and trunk. Deep tendon reflexes were normal, and bilateral pathological reflexes were absent. Finger-to-nose and heel-knee-shin tests were performed adequately, and coordination was grossly intact.

Routine hematological tests, hepatic and renal function panels, electrolytes, thyroid function, serum ammonia, lactate, creatine kinase, ceruloplasmin, and 24-hour urinary copper excretion were all within normal limits. The rheumatological workup revealed negative results for rheumatoid factor (RF), anti-cyclic citrullinated peptide (anti-CCP) antibodies, and antinuclear antibody (ANA) spectrum, with erythrocyte sedimentation rate (ESR) and C-reactive protein (CRP) also within normal ranges. Serum ferritin level was 35 μg/L (reference range: 13–150 μg/L). Lymphocyte subset analysis revealed a mildly decreased proportion of CD4-positive T lymphocytes. Lumbar puncture yielded clear and colorless cerebrospinal fluid (CSF) with an opening pressure of 140 mmH_2_O. CSF analysis showed mildly elevated white blood cell count at 16 × 10^6^/L (reference range: 0–15 × 10^6^/L), with lymphocyte predominance. CSF protein, glucose, and chloride levels were within normal limits. CSF bacterial culture, India ink staining, acid-fast staining, and tuberculosis-related assays were all negative.

The serum autoimmune encephalitis antibody panel (including anti-IgLON5, anti-NMDAR, anti-LGI1, anti-CASPR2, anti-GABABR, anti-DPPX, anti-GAD65, among 25 total) and CNS demyelinating antibodies (AQP4, MOG, GFAP) were all negative. Both serum and CSF oligoclonal bands (OCB) were negative. Serum GlyR antibodies were positive by cell-based assay (CBA) at a titer of 1:100 (**Figure 1**) with negative CSF findings, while tissue-based assay (TBA) on brain sections revealed weak positive staining in the hippocampus and cerebellum, and no other stiff-person spectrum disorder-related autoantibodies were detected. CSF metagenomic next-generation sequencing (NGS) revealed no pathogenic sequences. Tumor markers and whole-body tumor imaging screening (CT of the head, chest, and abdomen) showed no abnormalities.

**Figure 1:**
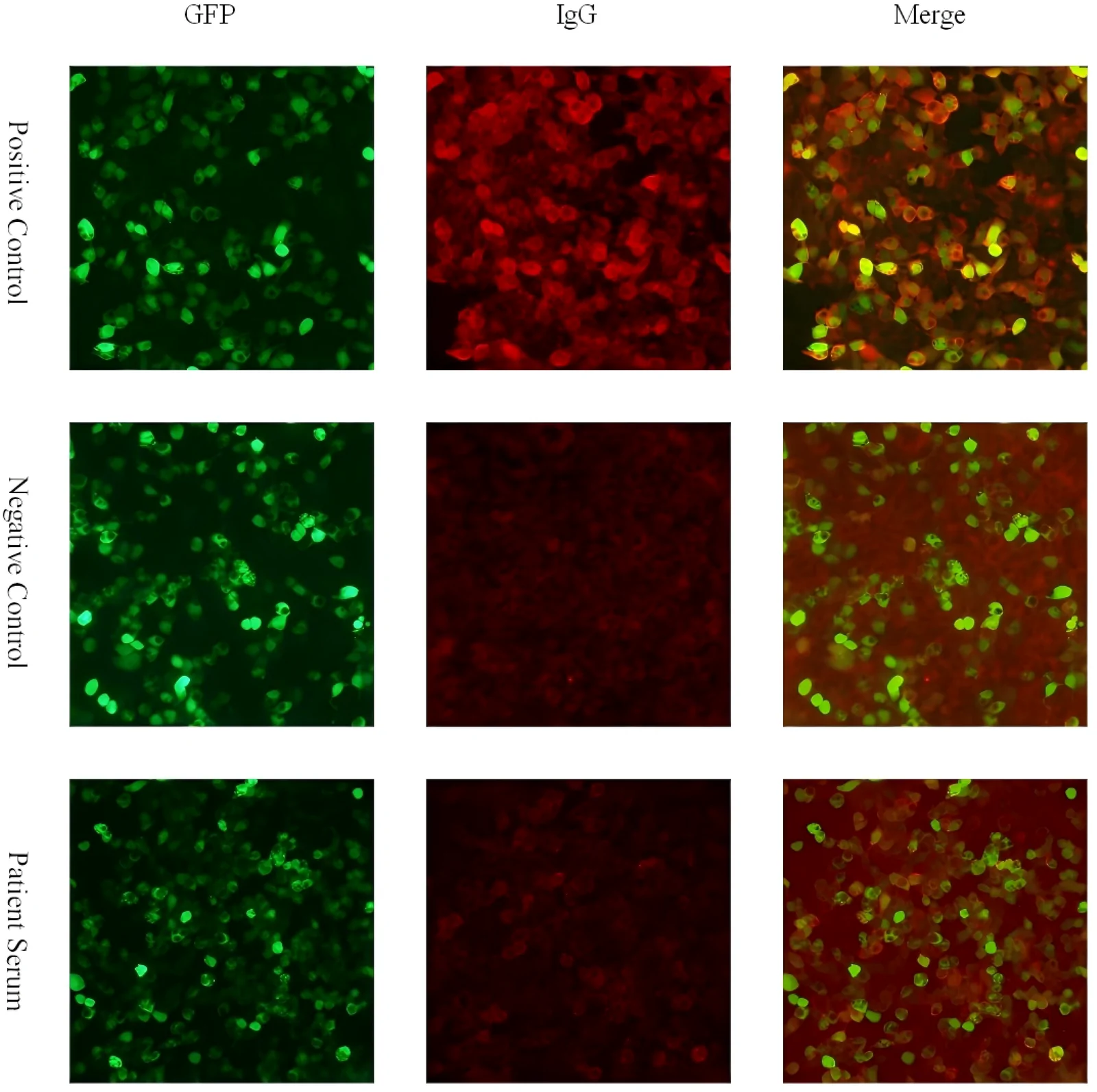
Detection of serum anti-GlyR antibodies by cell-based assay (CBA). Three rows represent positive control, negative control, and patient serum, respectively. Columns show GFP signal (green, indicating GlyR-transfected cells), IgG signal (red, indicating antibody binding), and merged images (GFP and IgG overlay). Co-localization of red IgG signal with green GFP-positive cells (yellow in merged images) confirmed the presence of anti-GlyR antibodies in patient serum at a titer of 1:100.

Video electroencephalography (VEEG) revealed no epileptiform discharges or background rhythm abnormalities. Electromyography (EMG) showed no typical continuous motor unit potentials or neurogenic damage; no myoclonic activity was captured during the examination. Cranial and whole-spine MRI with plain scan, DWI, and contrast enhancement demonstrated a patchy mild hyperintense lesion on T2-weighted imaging in the right brainstem, with no definite abnormal signals in other sequences (**Figure 2**).

**Figure 2:**
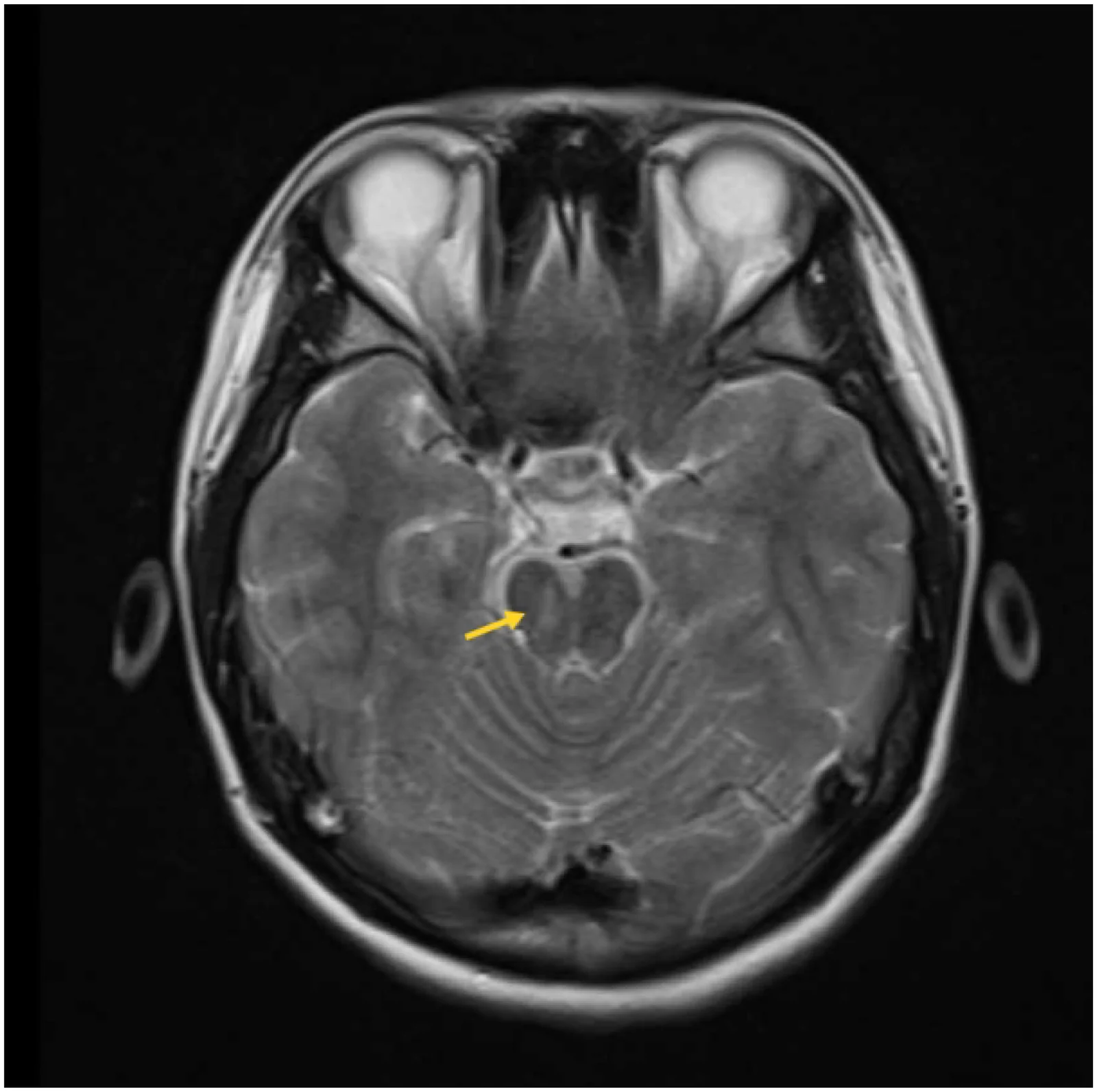
Brain and spinal cord MRI. A patchy mild T2-hyperintense lesion was observed in the right brainstem (yellow arrow), with no other abnormalities on cranial or whole-spine MRI, including DWI and contrast-enhanced sequences.

Based on the patient’s chronic progressive axial and limb rigidity, paroxysmal myoclonus, MRI findings of brainstem involvement, and positive serum anti-GlyR antibodies, and in accordance with the currently proposed diagnostic framework for stiff-person spectrum disorders (16), a clinical diagnosis of anti-GlyR antibody-positive PERM was established.

Following admission, the patient received intravenous immunoglobulin (IVIG, 400 mg/kg/day for 5 days) combined with methylprednisolone pulse therapy (30 mg/kg/day for 5 days), which was subsequently switched to oral corticosteroids with gradual tapering, along with long-term maintenance of clonazepam (4 mg/day, divided into two oral doses). At discharge, her muscle tone had substantially improved, and she was able to walk slowly with the assistance of a walker. At the 6-month follow-up, repeat testing showed that serum anti-GlyR antibody titers had decreased from 1:100 to negative. During the one-year regular follow-up period, she experienced gradual recovery of gait, maintained good cognitive function and academic performance, and her quality of life was largely unaffected, with no reported adverse drug reactions. The complete clinical timeline from disease onset to the last follow-up is shown in **Figure 3**. The patient continues to be followed up regularly.

**Figure 3:**
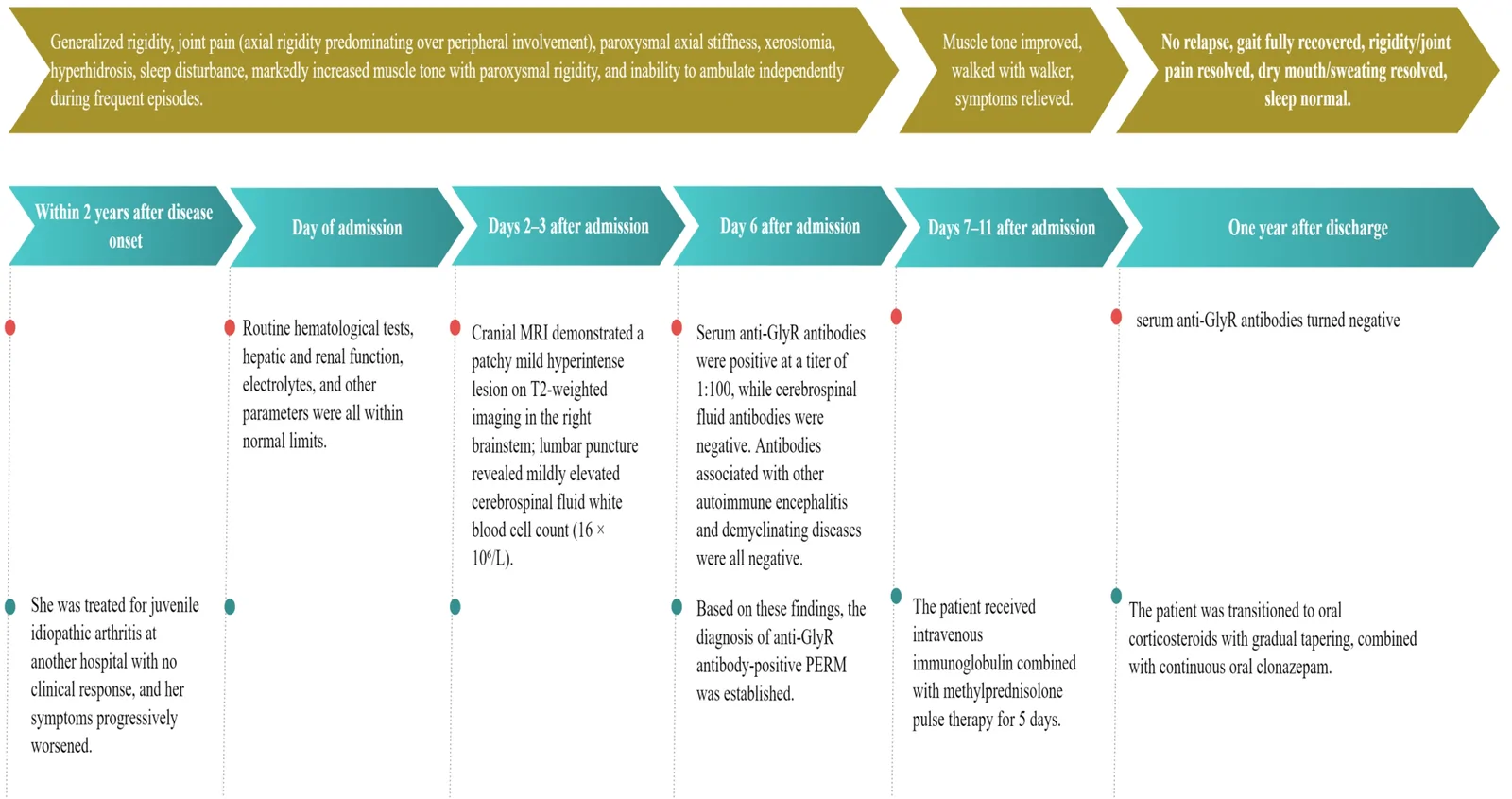
Complete clinical timeline of the patient from disease onset, diagnosis, treatment, to follow-up.

## Discussion

Stiff person spectrum disorders (SPSD) are a group of rare autoimmune diseases of the central nervous system, comprising four subtypes: classic SPS, partial SPS, classic SPS with cerebellar/brainstem involvement, and PERM (8). Among these, PERM is exceedingly rare in the pediatric population, with only 11 cases reported in children worldwide as of 2024 (9). In addition to the truncal and limb rigidity and painful spasms shared by all SPSD subtypes, PERM is further characterized by prominent brainstem dysfunction and autonomic disturbances (10). Of note, autonomic dysfunction in PERM manifests as a wide spectrum of symptoms including sleep disturbances, xerostomia, hyperhidrosis, and cardiovascular instability, which are thought to reflect brainstem involvement (2, 10). The pathogenesis of PERM is closely associated with autoantibodies, with anti-GlyR antibodies being the major pathogenic antibodies, while anti-glutamic acid decarboxylase (GAD) antibodies and anti-dipeptidyl peptidase-like protein 6 (DPPX) antibodies are relatively less common (2, 11).

GlyRs are pentameric chloride channels localized at postsynaptic membranes via anchoring proteins to mediate fast inhibitory neurotransmission, and their dysfunction due to anchoring disruption underlies related neurological disorders (12, 13). The distribution of GlyRs in the CNS has expanded from the traditional view of predominant α1-β heteromer expression in the spinal cord and brainstem to include the cerebellum and hippocampus (14). Anti-GlyR autoantibodies primarily target the GlyRα1 subunit and impair glycinergic inhibitory synaptic transmission through a dual mechanism of direct receptor blockade and receptor internalization (15). This mechanistic understanding not only explains the core symptoms of muscle rigidity and myoclonus in PERM patients but also provides a pathophysiological basis for the rapid clinical improvement and normalization of serum antibody titers observed in our patient following IVIG and corticosteroid therapy.

Currently, there are no unified diagnostic criteria for PERM. Clinical diagnosis primarily relies on the SPS spectrum disorder diagnostic framework proposed by Newsome and Johnson in 2022, which provides a practical reference for categorizing diagnoses based on the fulfillment of major and minor criteria (16). Although this framework has not been formally validated as a PERM-specific diagnostic standard, it offers a useful approach for clinical decision-making. Our patient met four major criteria (clinical presentation involving typical body regions, examination findings of truncal and limb rigidity with myoclonus, positive serum anti-GlyR antibodies, and exclusion of alternative diagnoses) and three minor criteria (autonomic dysfunction, mild CSF pleocytosis, and brainstem T2 hyperintensity on MRI), supporting a clinical diagnosis of PERM. We acknowledge that the clinical presentation of this case overlaps diagnostically with anti-GlyR antibody-associated SPSD, and that the chronic arthralgia-onset and two-year disease course represent atypical features of PERM. Nevertheless, considering the combination of axial rigidity, myoclonus, autonomic dysfunction, brainstem T2 hyperintensity on MRI, and positive serum anti-GlyR antibodies (titer 1:100), a clinical diagnosis of PERM remains the most reasonable interpretation based on the available evidence. However, the diagnostic journey of this patient was notably protracted. She presented with generalized joint pain and rigidity as the initial symptoms for two years and had been diagnosed with juvenile idiopathic arthritis at another hospital, although she lacked typical peripheral arthritis signs and showed poor response to therapy. The root cause of this diagnostic challenge lies in the highly nonspecific early clinical manifestations of PERM. In terms of differential diagnosis, according to the systematic review of juvenile idiopathic arthritis by Martini et al., under the ILAR classification framework, joint swelling, pain on motion, tenderness, and limited range of motion are the core objective parameters defining inflammatory articular involvement (17). Although our patient had joint pain, she consistently lacked such signs, and her RF, anti-CCP, ANA profile, ESR, and CRP were all normal, which was insufficient to establish a diagnosis of JIA. Inflammatory bowel disease (IBD)-associated arthropathy is one of the most common extraintestinal manifestations of IBD (mainly Crohn’s disease and ulcerative colitis) and falls within the spectrum of seronegative spondyloarthritis (18). Given that our patient had no chronic abdominal pain, diarrhea, hematochezia, or other gastrointestinal symptoms, with negative fecal occult blood test and normal serum ferritin (35 μg/L) and inflammatory markers (ESR, CRP), IBD-associated arthropathy could be reasonably excluded. Wilson’s disease is an autosomal recessive disorder of copper metabolism characterized by abnormal copper deposition in the liver, brain, cornea, and other tissues (19). Our patient had normal results for blood ammonia, lactate, ceruloplasmin, and 24-hour urinary copper quantification, with no corresponding clinical manifestations, thereby excluding this metabolic disorder. The root cause of this diagnostic challenge lies in the highly nonspecific early clinical manifestations of PERM. When the disease presents with joint pain rather than typical muscle rigidity, myoclonus, or brainstem signs, it is easily confused with rheumatic and immune-mediated diseases. In retrospect, several key clues should have directed the diagnosis toward a neurological origin earlier: the rigidity predominantly involved axial muscles with a paroxysmal pattern, and the joints themselves lacked typical signs of peripheral arthritis such as swelling, redness, or warmth. This pattern of “axial > peripheral, paroxysmal > persistent” rigidity was more consistent with a central nervous system disorder. Furthermore, the accompanying autonomic symptoms, including sleep disturbance, xerostomia, and hyperhidrosis, were difficult to explain by juvenile idiopathic arthritis but are common systemic involvement signals in PERM. Ultimately, positive serum anti-GlyR antibodies (titer 1:100) and brainstem T2 hyperintensity on MRI provided the key evidence for the clinical diagnosis.

To date, there are no unified treatment guidelines for PERM, and the management typically involves a comprehensive approach combining symptomatic and immunomodulatory therapies. The immunotherapy for PERM can follow the stepwise escalation strategy for SPSD: first-line therapies include IVIG or plasma exchange; if the response is inadequate, treatment can be escalated to second-line agents such as rituximab, mycophenolate mofetil, or azathioprine; refractory cases may be considered for cyclophosphamide or combination therapy (16). However, Dalakas, in a subsequent review, emphasized that the randomized controlled trial evidence for IVIG is derived primarily from studies in classic SPS rather than PERM, and that plasma exchange offers only transient benefits in the acute phase, while cyclophosphamide, mycophenolate mofetil, and azathioprine lack clear evidence of long-term benefit in PERM and are not recommended for routine use (20). Therefore, in clinical practice, IVIG should be prioritized as the foundational immunotherapy, with escalation reserved for carefully selected refractory cases. Furthermore, some case reports have suggested early initiation of immunotherapy in PERM patients and proposed combined maintenance therapy with oral corticosteroids to reduce the risk of relapse, although the optimal duration remains uncertain (21). For symptomatic treatment, benzodiazepines (such as clonazepam) are the cornerstone for relieving muscle rigidity and spasms; when benzodiazepines are insufficient, other agents such as baclofen, tizanidine, or gabapentin may be considered in combination (22). Additionally, approximately 20% of PERM cases are associated with paraneoplastic syndromes, and therefore tumor surveillance for at least two years is recommended for these patients (10, 22).

## Conclusion

We report a rare pediatric case of anti-GlyR antibody-positive PERM with an atypical presentation of chronic joint pain and axial rigidity that had been initially attributed to juvenile idiopathic arthritis, although the patient lacked typical peripheral arthritis signs, which underscores the importance of considering this disease in children with unexplained musculoskeletal symptoms and autonomic dysfunction. This case highlights the critical role of early recognition, prompt serum and cerebrospinal fluid antibody testing, comprehensive neurological imaging, and appropriate immunomodulatory therapy in achieving favorable outcomes and preventing life-threatening complications, even in the setting of delayed diagnosis. Furthermore, as the global awareness of PERM increases, clinicians should maintain a high index of suspicion for this condition in pediatric patients presenting with nonspecific axial rigidity and autonomic features and implement standardized diagnostic algorithms to avoid misdiagnosis. Future studies should further investigate the full clinical and serological spectrum of pediatric PERM and develop more refined diagnostic criteria and treatment guidelines to improve early detection and long-term prognosis in this rare but potentially fatal disorder.

## Data Availability

The original contributions presented in the study are included in the article/Supplementary Material. Further inquiries can be directed to the corresponding author.
